# Prediction of molecular mechanism of processed ginseng in the treatment of heart failure based on network pharmacology and molecular docking technology

**DOI:** 10.1097/MD.0000000000036576

**Published:** 2023-12-08

**Authors:** Tingting Dai, Jiyu Gong, Shuying Liu

**Affiliations:** a School of Pharmaceutical Sciences, Changchun University of Chinese Medicine, Changchun, China; b Jilin Ginseng Academy, Changchun University of Chinese Medicine, Changchun, China.

**Keywords:** heart failure, molecular docking, network pharmacology, processed *Panax ginseng* C.A. Mey. products

## Abstract

**Background:**

Heart failure (HF) is the most common cardiovascular disease in clinics. Processed *Panax ginseng* C.A. Mey. Products have significant therapeutic effects on HF. Therefore, it is of great significance to explore the mechanism of action of Processed *Panax ginseng* C.A. Mey. Products in the treatment of HF.

**Methods:**

The saponin-like constituents of 3 different ginseng preparations were characterized by UPLC/QE-MS and the identified saponin constituents were subjected to network pharmacological analysis. Protein–protein interaction analyses of the targets of different ginseng preparations for the treatment of heart failure (HF) were performed using the STRING database, Gene Ontology enrichment analyses and Kyoto Encyclopedia of Genes and Genomes pathway enrichment analyses were performed using the DAVID database, and the results of the network pharmacological analyses were validated using the Autodock software. Finally, the relative quantitative content of 5 major ginsenosides in 3 processed ginseng products was evaluated.

**Results:**

A total of 40 saponin compounds were identified based on mass spectrometry data. Network pharmacology and molecular docking analyses were used to predict the major targets of these sapions compounds and the key pathways mediating their anti-HF effects. After conducting a thorough screening, the study identified 5 primary ingredients of ginseng products ginsenoside Rh4, ginsenoside Rk3, ginsenoside Rk1, ginsenoside Rg5, and ginsenoside CK that can potentially target 22 essential proteins: EGFR, AKT1, ERBB2, STAT3, TNF, ESR1, MTOR, HRAS, MMP9, and PIK3CA, etc. Additionally, the Kyoto Encyclopedia of Genes and Genomes pathway analysis revealed that ginseng products can be beneficial in treating HF by interacting with pathways such as the PI3K-Akt signaling pathway, the TNF signaling pathway, the mTOR signaling pathway, and others.

**Conclusion:**

The present study revealed that the treatment of HF with different processed ginseng products may be related to the regulation of the PI3K-Akt signaling pathway, TNF signaling pathway, apoptosis pathway, mTOR signaling pathway, etc, and that the key active ingredients may be concentrated in black ginseng, which provides a theoretical basis and direction for the further study of the mechanism of action of ginseng. This provides a theoretical basis and research direction for further in-depth study of its mechanism of action.

## 1. Introduction

Heart failure (HF) is a complex clinical syndrome caused by the progression of various cardiac diseases to the severe stage. It has become the leading cause of death worldwide, often associated with dyspnea, fluid retention, reduced exercise tolerance, etc, and HF accounts for 32% of all deaths worldwide, with a low cure rate. Patients with underlying heart disease are more likely to develop HF.^[1]^ In recent decades, Western medicine has made great strides in improving the prognosis and reducing the morbidity and mortality of patients with HF. However, the “single-gene-single-target” treatment mode of Western drugs also brings certain side effects, which limits its clinical application in some aspects. Chinese medicine has accumulated rich clinical experience in the prevention and treatment of HF, especially in controlling symptoms, improving cardiac function, and enhancing quality of life.

Ginseng (*Panax ginseng* C.A. Mey.) has been a valuable medicinal material used in traditional oriental medicine for thousands of years and is now widely used as a healthy food in East Asia and worldwide. Furthermore, not only raw sun-dried ginseng but also several processed ginseng products have been used in the food industry and herbal markets.^[2]^ According to the different processing methods, there are 3 processed ginseng products, raw sun-dried ginseng (DG), red ginseng (RG), and black ginseng (BG). Ginsenosides are considered to be the main active substances of ginseng and ginseng products. Previous studies have demonstrated that ginseng has significant positive inotropic effects, which can significantly enhance myocardial contractility, improve ventricular remodeling, bring the hemodynamics of patients with heart failure (HF) to a stable state, and contribute to the improvement of cardiac function and structure.^[3]^ Wan^[4]^ found that black ginseng was stronger than RG and ginseng against HF. Cao^[5]^ showed that based on the TGF-β/Smad signaling pathway, black ginseng decoction had a stronger protective effect than white ginseng decoction on cardiac hypertrophy rats. Liu^[6]^ found that Rg5 significantly reduced cardiac dilatation and venous congestion and increased cardiac output and heart rate. It has also been shown that Rg5 improves metabolic disorders and cardiac dilatation in zebrafish with HF. Dai^[7]^ found that TSBG has an excellent therapeutic effect on DOX-induced HF in rats, probably by regulating the Akt/mTOR autophagy signaling pathway, resulting in the improvement of taurine and hypotaurine metabolism, arachidonic acid metabolism and sphingolipid metabolism, which may provide a reference for elucidating the potential mechanism of action of TSBG against HF. However, the key active components, targets of action and biological mechanisms of different processed products of ginseng to comprehensively regulate and improve the whole of HF from the overall network level are not clear.

Network pharmacology is a research methodology that integrates molecular docking, construction of drug-disease target networks, and network characterization for the holistic regulation of multiple targets, which is instructive for the development of new drugs as well as elucidating the molecular mechanisms behind drugs.^[8]^ It can systematically present the interrelationship between drugs, compounds, targets and diseases. It has become an important method to study traditional Chinese medicine (TCM) formulas with “multi-component, multi-pathway and multi-target.” This study intends to explore the bioactive components and mechanism of action of ginseng-processed products for HF at a systemic level by integrating network pharmacology and molecular docking techniques, to provide a theoretical basis and research direction for subsequent clinical and basic studies.

## 2. Materials and methods

### 2.1. Sample collection

DG, RG, and BG are 5 years old, Changbai county origin of ginseng processed products. In 2020, all proceed ginseng was purchased from Jilin Cidayan Traditional Chinese Medicine Distribution Co., Ltd.

### 2.2. Identification of ginsenosides

#### 2.2.1. Chemicals and reagents.

HPLC-grade acetonitrile and methanol were purchased from Fisher Scientific (USA). HPLC-grade formic acid was purchased from TEDIA (USA). Methanol, and alcohol (Analytical grade) were purchased from Beijing Chemical Plant (China). The ginsenoside standards used in Shanghai Yuanye Biotechnology Co., Ltd (Shanghai, China). Rg1 (22427-39-0), Re (52286-59-6), Rb1 (41753-43-9), Ro (34367-04-9), Rc (11021-14-0), Rd (52705-93-8), Rg3 (14197-60-5), CK (39262-14-1), Rk1 (494753-69-4). The purities of these compounds were more than 98%.

#### 2.2.2. Sample preparation.

The DG, RG, and BG samples were crushed and sieved to obtain fine powder for later use. Fine powder (100 mg) from DG, RG, and BG samples was suspended in 25 mL of 70% (v/v) methanol, and ultrasonically extracted for 60 minutes at 55°C. After centrifugation (13,500 rpm, 5 minutes), the solution was filtered by using a syringe filter (0.22 µm) and injected into the UPLC system.

#### 2.2.3. UPLC-QE-OrbitrapMS conditions.

For metabolite profiling of the processed ginseng products, we used a previously constructed method based on UPLC-QE-OrbitrapMS.^[9]^

Chromatographic separation was performed on an Ultimate 3000 system (Dionex, Sunnyvale, CA) coupled with Waters BEH RP-18 column (2.1 × 100 mm, 1.7 μm; Waters, Milford, MA). The column temperature was maintained at 30 °C, and the mobile phases A and B were water with 0.1% formic acid and acetonitrile, respectively. The gradient elution program was as follows: 0 to 5 minutes (75%A), 5 to 8 minutes (75–64%A), 8 to 15 minutes (64–52%A), 15 to 20 minutes (52–30%A), 20 to 25 minutes (30–10%A), 25 to 28 minutes (10%A), 28 to 28.1 minutes (10–75%A), 28.1 to 34 minutes (75%A); the flow rate was set at 0.2 mL/min, and the sample injection volume was 1 µL for each run.

Mass spectrometric detection was carried out on a Q-Exactive Orbitrap/MS (Thermo, San Jose, CA) equipped with an ESI ion source operated in the positive or negative ion mode. The parameters of the ion source were set as follows: sheath gas flow of 40 Arb, aux gas flow of 12 Arb, and sweep gas flow of 1 Arb, e S-Lens RF level was 55%. Capillary voltage was set to +3.5 kV or −3.5 kV with a capillary temperature of 333 °C and an aux gas heater temperature of 317 °C. The high-accuracy MS data were collected from m/z 100 to 1500 Da in positive or negative ion mode.

### 2.3. Network pharmacology

#### 2.3.1. Identification of ginsenosides components and prediction of targets.

Using the Swiss Target Prediction platform (http://www.swisstargetprediction.ch) to predict identified different processed products of ginseng ginsenosides compounds of potential targets.

#### 2.3.2. Target screening for HF.

Using OMIM database (https://omim.org/), DISGENET database (https://www.disgenet.org/)^[10]^ and Drug bank database (https://www.drugbank.com/), “Heart failure” was used as the search keyword, and SOCRE > 0.7^[11]^ was used as the screening condition in the DISGENET database to retrieve the targets related to HF disease. After the integration of the disease database, the repeated targets were eliminated for screening.

#### 2.3.3. Candidate target library and protein interaction network (PPI) construction.

The target sites of ginsenosides identified were mapped to the target sites of HF, and the Venn diagram was drawn to visualize the data. The intersection of component targets and disease targets was calculated to establish a candidate target library. The candidate targets were imported into the String database (https://cn.string-db.org/), and relevant parameters were set. The species was selected as “Homo sapiens,” and the confidence score represented the PPI degree, and the confidence was set as 0.4. The generated files were imported into Cytoscape software (3.9.1)^[12]^ to screen the top 50 hub genes under MCC, and the PPI network diagram was visualized. At the same time, the network topology analysis is carried out to calculate the “degree,” “Betweenness centrality,” and “Closeness Centrality” values, and then to obtain the core target.

#### 2.3.4. GO enrichment analysis and KEGG signaling pathway analysis.

Gene Ontology enrichment (GO) and Kyoto Encyclopedia of Genes and Genomes pathway analysis (KEGG) were performed on the targets using the Mescape database (https://metascape.org/gp/index.html).^[13]^ GO enrichment analysis selected biological process (BP), molecular function (MF) and cellular component (CC). Second, choose *P* < .01, the result of the database access data import microscopic letter to the platform (http://www.bioinformatics.com.cn/), which will access visual analysis, generate KEGG bubble chart and GO histogram analysis.

#### 2.3.5. Mapping of “disease-component-target-pathway” network and screening of active components.

The “disease-component-target-pathway” network was constructed by Cytoscape3.9.1 software, and the potential targets of ginseng, RG, and black ginseng and the disease targets of HF were further compared, and the network characteristics were analyzed by Netweek analysis, a plug-in of Cytoscape3.9.1 software. To explore the pharmacological mechanism of ginseng, RG, and black ginseng saponins in the treatment of HF.

### 2.4. Molecular docking

To further evaluate the interaction between target proteins and compound molecules, molecular docking between target proteins and compound molecules was performed using AutoDock Vina molecular docking software. The core protein structure of the PPI network was downloaded from the PDB database (http://www.rcsb.org/), and its ligands and water molecules were removed with Pymol. The 3D structure information of protein and compound structure was uploaded, and then molecular docking was carried out to obtain the docking results of the target protein and compound molecules.^[14]^

### 2.5. Relative content of core components

The core components of ginsenoside in the treatment of HF were obtained through network pharmacology combined with molecular docking. The relative contents of the core components in the different processed products of 3 kinds of ginseng were compared by boxplot.

## 3. Results

### 3.1. UPLC-Q Exactive Orbitrap-MS/MS identification

**Figure 1.**
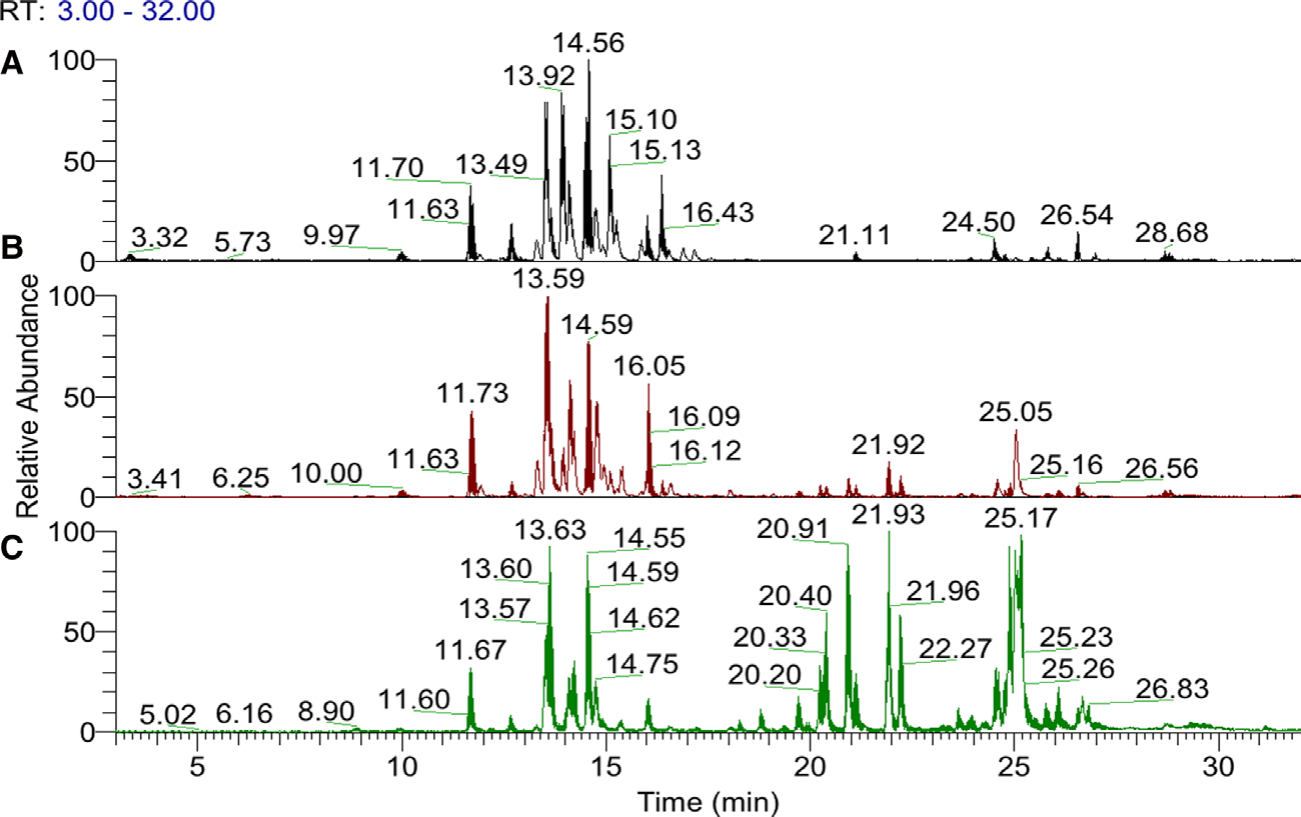
The base peak intensity (BPI) chromatogram of diverse metabolites from (A) raw sun-dried ginseng, (B) red ginseng, (C) black ginseng.

70% (v/v) methanol was used to extract ginsenosides and other metabolites from the 3 samples including DG, RG, and BG. The extracts were then subjected to UPLC-QE-Orbitrap MS in positive and negative ion mode. Figure 1 shows the representative base peak intensity chromatograms of different metabolites from DG, RG, and BG. UPLC effectively separated different metabolites in 34 minutes. The intensities of several peaks were different depending on the different samples. For example, compared to other products, the elution peak intensity of BG was higher at 20 to 30 minutes, DG had more than other products at 12 to 18 minutes in the base peak intensity chromatogram. These results indicated that different processing methods could change the metabolic components of different ginseng products. This section may be divided by subheadings. It should provide a concise and precise description of the experimental results, their interpretation, as well as the experimental conclusions that can be drawn.

After method analysis, a total of 40 ginsenosides were identified in ginseng processed products, of which 9 ginsenosides were qualitatively identified by standard products, and the remaining 31 ginsenosides were obtained by literature review, as shown in Table 1.^[15–19]^ Ginseng produces trace amounts of rare saponins during processing, which are the products of deglycosylation, dehydration and oxidation at the C-20 position of the original ginseng diol (triol) type saponin.

**Table 1:** Ginsenosides mass spectrometry data from 3 processed Panax ginseng C.A. Mey. products such as raw sun-dried ginseng, red ginseng, and black ginseng.

RT	Compound	Formula	Theoretical mass (m)	Experimental mass (m)	Error value (ppm)	Fragment ion (m/z)	Adducts	Precursors (m/z)
3.31	mRe	C_51_H_84_O_21_	1032.5505	1032.5377	0.6	945.5172, 783.4734, 637.4213, 475.3653	-H	1031.5413
3.41	Re2或Re3	C_48_H_82_O_19_	962.545	962.533	0.58	799.4726, 637.4315, 475.3780	-H	961.5354
8.84	Rh1	C_36_H_62_O_9_	638.4394	638.4292	−2.92	475.3587, 391.2654	-H	637.4321
9.89	20 (R)-Rh1	C_36_H_52_O_9_	638.4394	638.4292	−2.98	475.3698, 391.2713	-H	637.421
11.15	Rg1	C_42_H_72_O_14_	800.4922	800.4814	−0.5	637.4357, 475.3695, 391.2801	-H	799.463
12.66	Rk3	C_36_H_60_O_8_	620.4288	620.4188	−3.1	457.3572	-H	619.419
13.5	R4	C_59_H_100_O_27_	1240.6452	1240.627	−1.7	1107.5881, 945.5145, 783.4778, 621.4297,		1239.5983
13.5	Rb1	C_54_H_92_O_23_	1108.6029	1108.5873	−1.05	945.5189, 783.4812, 621.4289, 459.3795	-H	1107.5875
13.5	Re	C_48_H_82_O_18_	946.5501	946.5356	−2.36	783.4782, 637.4210, 475.3699	-H	945.5197
13.52	F4	C_42_H_70_O_12_	766.4867	766.4741	−3.42	765.4702, 603.3318, 441.2485	+COOH,-H	811.4726
13.6	Noto R2	C_41_H_70_O_13_	770.4816	770.47	−2.06	637.4213, 475.3678, 391.2830	-H	769.4721
13.63	Rg2	C_42_H_72_O_13_	784.4973	784.4846	−3.24	637.4228, 619.4050, 475.3705, 391.2801	-H	783.4783
13.7	mRa2	C_61_H_100_O_29_	1296.635	1296.6169	−0.94	1209.6075, 1077.5518, 945.5183, 783.4821, 621.4252,	-H	1295.6210
13.9	mRb1	C_57_H_94_O_26_	1194.6033	1194.5874	−0.29	1107.5815, 945.5297, 783.4807, 621.4251, 459.3793	-H	1193.5712
14.11	Rc	C_53_H_90_O_22_	1078.5924	1078.5771	−1.15	945.5187, 783.4806, 621.4291, 459.3795	-H	1077.5885
14.17	Ra2	C_58_H_98_O_26_	1210.6346	1210.618	−0.68	1077.5549, 945.5195, 783.4800, 621.4293	-H	1209.6081
14.22	Rh4	C_36_H_60_O_8_	620.4288	620.419	−2.75	457.3658	-H	619.4183
14.47	mRa1	C_61_H_100_O_29_	1296.635	1296.6165	−1.23	1209.6103, 1077.5813, 945.5145, 783.4792, 621.4223	-H	1295.635
14.47	mRc	C_56_H_92_O_25_	1164.5928	1164.5776	−0.03	1077.5813, 945.5187, 783.4787, 621.4310, 459.3734	-H	1163.5721
14.54	Ro	C_48_H_76_O_19_	956.4981	956.486	0.33	793.4325, 613.3676	-H	955.4679
14.55	Ra1	C_58_H_98_O_26_	1210.6346	1210.6205	1.33	1077.5823, 945.5135, 783.4785, 621.4263, 459.3821	-H	1209.6213
14.69	F3	C_41_H_70_O_13_	770.4816	770.4691	−3.24	621.4249, 475.3751, 391.2816	-H	769.4714
14.75	Rb2	C_53_H_90_O_22_	1078.5924	1078.5749	−3.22	945.5143, 783.4762, 621.4259, 459.3792	-H	1077.583
14.93	Rb3	C_53_H_90_O_22_	1078.5924	1078.5771	−1.78	945.5163, 783.4795, 621.4276, 459.3671	-H	1077.5553
15.07	mRb2	C_56_H_92_O_25_	1164.5928	1164.5781	0.41	1077.5823, 945.5238, 783.4835, 621.4316, 459.3833	-H	1163.5813
15.83	Rf	C_42_H_72_O_14_	800.4922	800.4817	−0.06	637.4216, 475.3704, 391.2727	-H	799.4643
15.86	mRb3	C_56_H_92_O_25_	1164.5928	1164.5777	0.01	1077.5851, 945.5152, 783.4769, 621.4276, 459.3619	-H	1163.5305
16.03	Rd	C_48_H_82_O_18_	946.5501	946.536	−1.88	783.4779, 621.4272, 459.3703	-H	945.5164
16.67	F1	C_36_H_62_O_9_	638.4394	638.4293	−2.83	475.3693, 391.2839	-H	637.4215
17.17	mRd	C_51_H_84_O_21_	1032.5505	1032.5375	0.43	945.5172, 837.4123, 537.4146, 459.3725	-H	1031.5273
21.05	Rs3	C_44_H_74_O_14_	826.5079	826.4946	−3.09	783.4782,621.4410,459.3791,375.2914	-H	825.4984
21.58	Noto R1	C_47_H_80_O_18_	932.5345	932.5232	0.83	799.4632, 637.4301, 475.3710, 391.2818	-H	931.5239
22.4	20(R)Rs3	C_44_H_74_O_14_	826.5079	826.4967	−0.54	783.4753,621.4421,459.3782,375.3013	-H	825.4913
22.69	F2	C_42_H_72_O_13_	784.4973	784.4849	−2.75	621.4263, 459.3781	-H	783.4770
23.21	20(R)Rg3	C_42_H_72_O_13_	784.4973	784.4852	−2.42	621.4287, 459.3793	-H	783.4795
25.28	Rg3	C_42_H_72_O_13_	784.4973	784.4878	0.85	621.4292, 459.3796	-H	783.4794
24.81	Rk1	C_42_H_70_O_12_	766.4867	766.4739	−3.75	603.4200, 537.3837,	-H	765.4711
26.82	Rg5	C_42_H_70_O_12_	766.4867	766.4739	−3.7	603.4203, 439.3557	-H	765.4692
30.56	Rh2	C_36_H_62_O_8_	622.4445	622.4366	0.36	443.3245,163.1269	-H	621.4179
31.86	Compound k	C_36_H_62_O_8_	622.4445	622.4365	0.15	459.3781, 375.2854	-H	621.4175

### 3.2. Constructing the “TCM-disease” intersection target database

**Figure 2.**
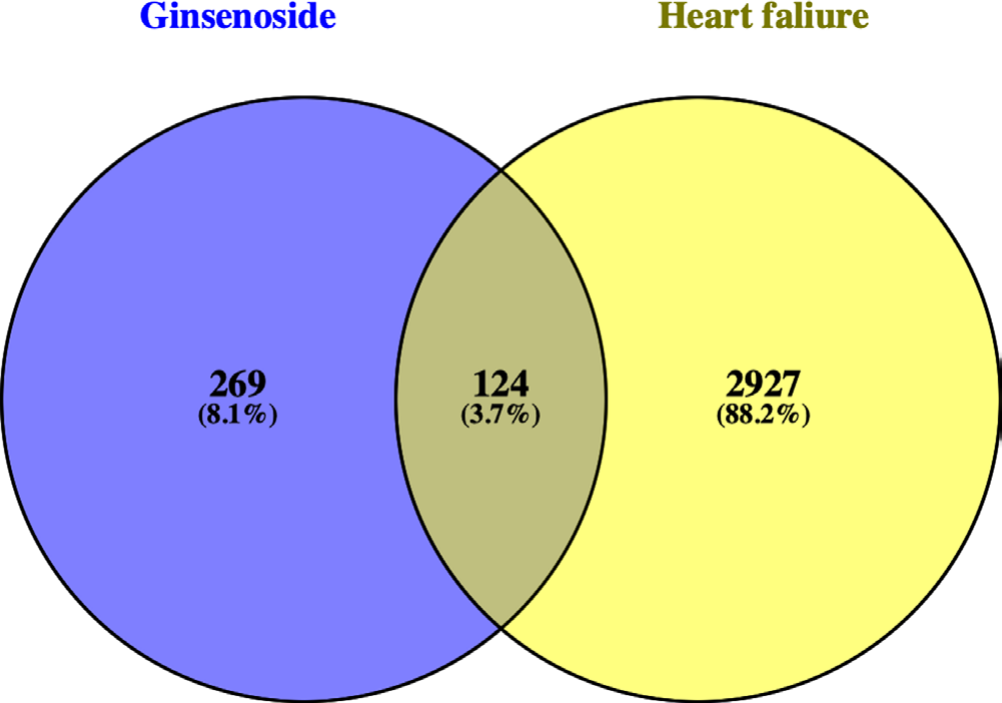
Venny diagram of ginsenoside and heart failure.

The targets were collected from SwissTarget Prediction, OMIM, Drug Bank and DISGENET database, and 3051 targets were finally obtained after deleting the duplicate targets. Finally, 401 related targets of ginsenosides and 3051 disease-related targets were obtained. The Venny diagram (Fig. 2) was constructed, and 124 intersection targets of the 2 were obtained as candidate targets.

### 3.3. Protein–protein interaction (PPI) network construction

**Figure 3.**
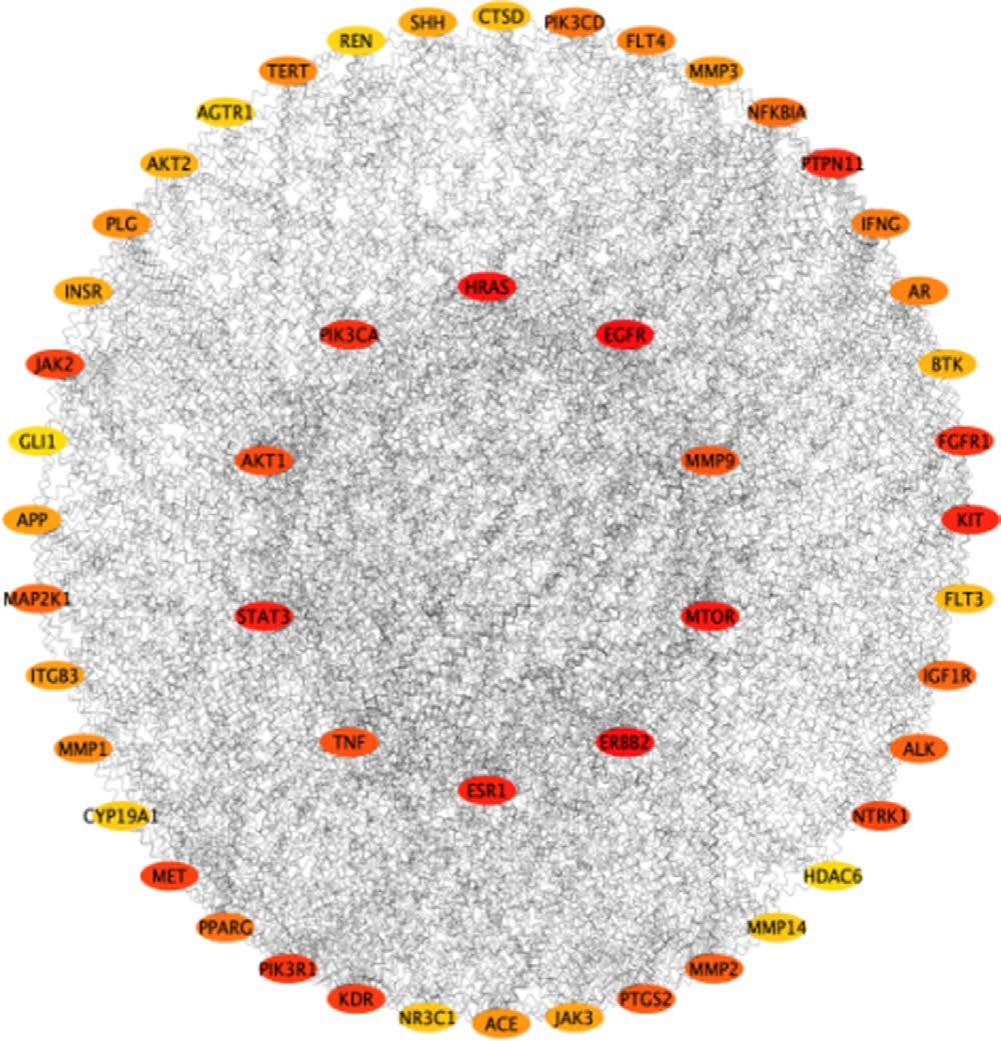
Hub genes protein–protein interaction (PPI) network.

Cytoscape software was used to screen the top 50 hub genes from candidate target sites according to the method in Section 4.3.3, and the PPI network diagram was visualized (Fig. 3). As shown in the figure, the PPI network contains 50 nodes and 598 interaction relationships. The threshold value of a degree, betweenness centrality and closeness centrality is taken as screening, and the 22 obtained intersection targets are taken as core targets, and the results are shown in Table 2. The “Degree” value is higher, shows that its associated node, the more important degree is higher. EGFR, AKT1, ERBB2, STAT3, TNF, ESR1, mTOR, HRAS, MMP9, and PIK3CA ranked high (degree > 33), indicating that these targets may play an important role.

**Table 2:** The core targets of ginsenoside for HF treatment and the topological parameters.

No.	Target	Degree	Betweenness centrality	Closeness centrality
1	EGFR	46	0.052	0.942
2	AKT1	44	0.051	0.907
3	ERBB2	43	0.042	0.891
4	STAT3	42	0.041	0.875
5	TNF	42	0.045	0.875
6	ESR1	40	0.034	0.845
7	MTOR	39	0.032	0.831
8	HRAS	37	0.021	0.803
9	MMP9	34	0.021	0.766
10	PIK3CA	33	0.017	0.754
11	PTGS2	31	0.014	0.731
12	MMP2	31	0.015	0.731
13	KDR	31	0.013	0.731
14	PIK3R1	31	0.014	0.731
15	PTPN11	30	0.012	0.721
16	JAK2	30	0.014	0.721
17	KIT	28	0.008	0.700
18	IFNG	27	0.010	0.690
19	PPARG	27	0.012	0.690
20	MET	26	0.008	0.681
21	IGF1R	24	0.005	0.662
22	FGFR1	24	0.005	0.662

### 3.4. GO biological process enrichment analysis

**Figure 4.**
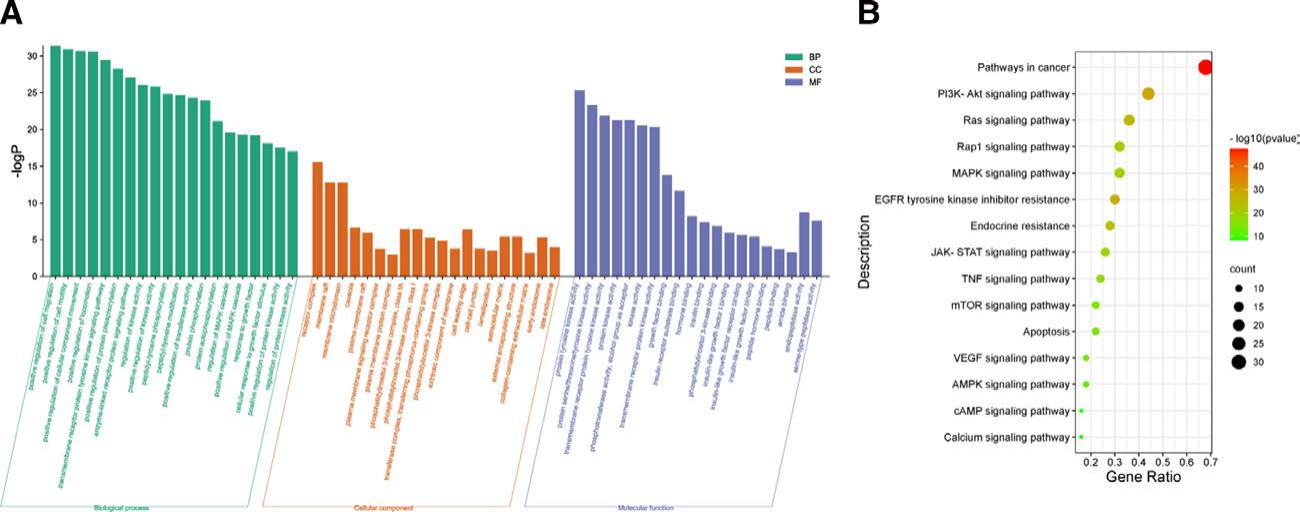
(A) GO and (B) KEGG pathway analysis of hub genes.

To investigate the biological functions of the target genes, 3 GO functional analyses were performed on 50 hub genes, including BP, CC, and MF, and the results with significant enrichment were visualized (*P* < .01), as shown in Figure 4A. BP mainly includes positive regulation of cell migration, positive regulation of cell motility, transmembrane receptor protein tyrosine kinase signaling pathway, etc.

CC mainly involves receptor complex, phosphatidylinositol 3-kinase complex, class I, membrane microdomain, etc. MF mainly involves protein tyrosine kinase activity, protein serine/threonine/tyrosine kinase activity, transmembrane receptor protein tyrosine kinase activity, etc. KEGG pathway enrichment analysis can explain which pathway ginsenoside acts on in the treatment of HF. 15 The pathways (*P* < .01) with significant enrichment and high disease relevance were screened out from 50 hub genes as shown in Figure 4B and Table 3. Major pathways include pathways in cancer, PI3K-Akt pathway, TNF pathway, apoptosis pathway, mTOR pathway, etc.

**Table 3:** KEGG pathway analysis of hub genes.

hsa number	Pathway name	Log *P*	Number of enriched targets
hsa05200	Pathways in cancer	−47.55	34
hsa04151	PI3K-Akt signaling pathway	−28.95	22
hsa04014	Ras signaling pathway	−25.19	18
hsa04010	MAPK signaling pathway	−19.80	16
hsa01521	EGFR tyrosine kinase inhibitor resistance	−27.03	15
hsa04630	JAK-STAT signaling pathway	−18.25	13
hsa04668	TNF signaling pathway	−18.40	12
hsa04210	Apoptosis signaling pathway	−15.48	11
hsa04150	mTOR signaling pathway	−14.81	11
hsa04370	VEGF signaling pathway	−15.29	9
hsa04152	AMPK signaling pathway	−12.40	9
hsa04024	cAMP signaling pathway	−8.52	8
hsa04020	Calcium signaling pathway	−8.24	8
hsa01522	Endocrine resistance	−23.33	14
hsa04015	Rap1 signaling pathway	−22.17	16

### 3.5. Component-target-disease-pathway network diagram

**Figure 5.**
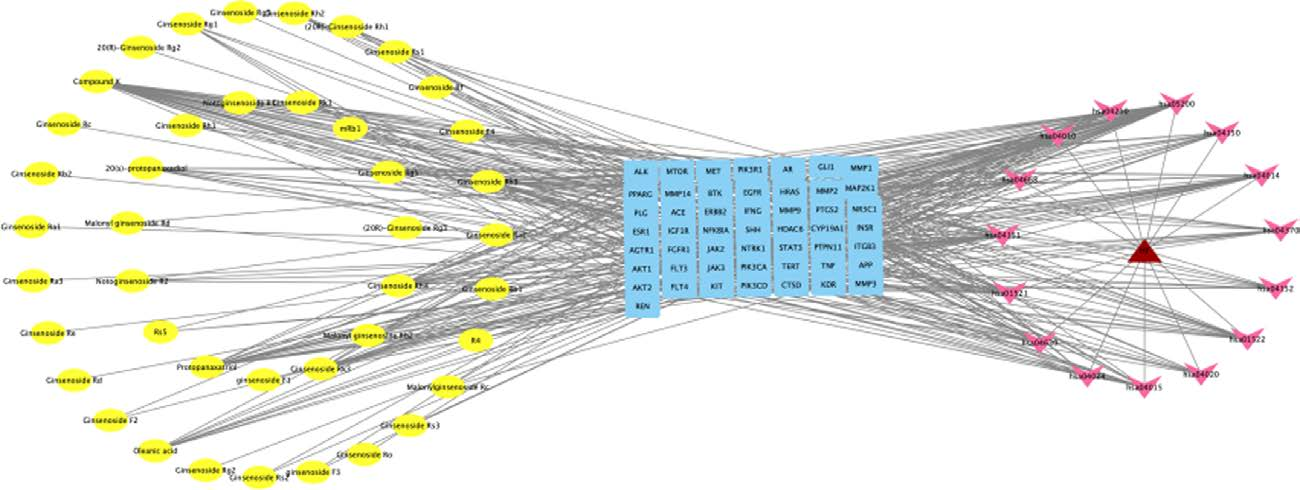
Compounds-Targets-Disease-Pathways network.

To further investigate the mechanism of action of ginsenosides from different ginseng derivatives in the treatment of HF, a component-target-disease-pathway network diagram was constructed using Cytoscape software (Fig. 5), which visually shows the relationship between each part. The network consists of 182 nodes and 556 edges, including HF (red triangular nodes), ginsenosides derived from various ginseng products (yellow circular nodes), 50 targets (blue rectangular nodes), and 15 pathways (pink arrow nodes). The closely related nodes were filtered by degree value. Ginsenoside Compound K, Ginsenoside Rk3, Ginsenoside Rh4, Ginsenoside Rk1, Ginsenoside Rg5, Ginsenoside F4, and Ginsenoside Rh1 may be important components in the treatment of HF. STAT3, PIK3CA, MAP2K1, PIK3CD, AKT1, MTOR, EGFR, AKT2, PIK3R1, and KDR rank high, suggesting that they are important targets for disease treatment. Pathways in cancer, PI3K-AKT pathway, Ras pathway, apoptosis pathway, and mTOR pathway are important signaling pathways for the treatment of disease.

### 3.6. Molecular docking

**Figure 6.**
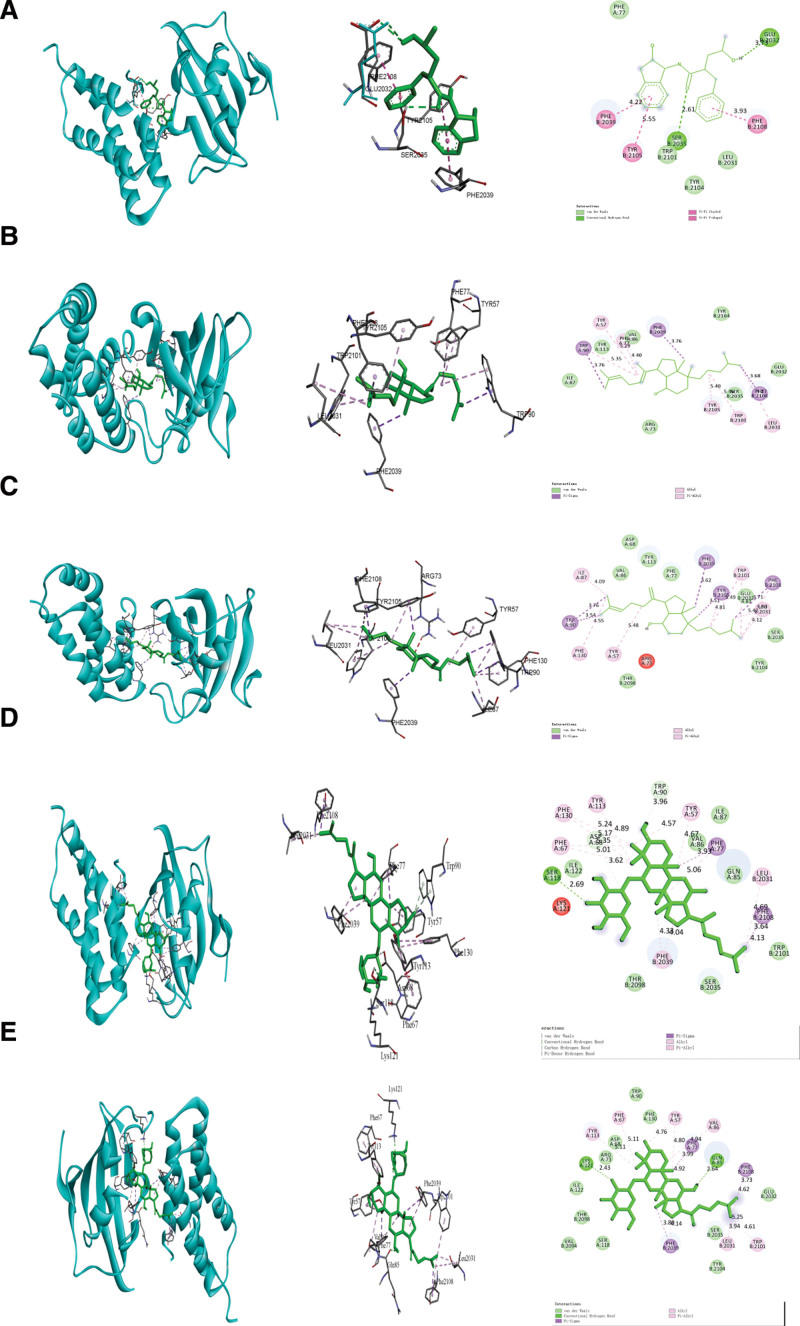
The represented results for the action mode of 5 active compounds with mTOR protein using molecular docking. (A) mTOR-Compound K, (B) mTOR-Ginsenoside Rg5, (C) mTOR-Ginsenoside Rk1, (D) mTOR-Ginsenoside Rk3, mTOR-Ginsenoside Rh4 (E).

The core active components of various *Panax ginseng* C.A.Mey products were screened and their core targets were docked. The lower the energy when the molecular docking ligand binds to the acceptor in a stable conformation, the more likely it is to occur. The results showed that ginsenoside Rh4 and ginsenoside Rk3 had the strongest binding force with mTOR (−11.5 kcal/mol), and the core components were found to have a strong binding force with mTOR (Fig. 6). The molecular docking score is shown in Table 4. These components play an important role in the treatment of HF in various derived products of ginseng, which can be used as a research object for the extraction of monomeric ginsenosides for the treatment of cardiovascular diseases in the future.

**Table 4:** Molecular docking results. Docking score are expressed as kcal·mol^-1^.

Chemical Compound	EGFR	AKT1	ERBB2	STAT3	TNF	ESR1	MTOR	HRAS	MMP9	PIK3CA
Compound K	−7.1	−6.0	−8.8	−6.4	−5.2	−7.3	−9.8	−7.9	−9.4	−8.3
Ginsenoside Rh4	−8.5	−6.8	−6.8	−7.4	−5.5	−6.8	−11.5	−7.3	−8.5	−7.6
Ginsenoside Rk3	−8.6	−6.3	−6.8	−6.9	−4.9	−6.8	−11.5	−8.5	−8.6	−7.7
Ginsenoside Rg5	−6.4	−5.5	−4.2	−6.0	−4.6	−6.8	−9.4	−6.8	−7.2	−7.4
Ginsenoside Rk1	−6.5	−4.9	−4.5	−5.5	−4.3	−6.9	−9.5	6.9	−7.2	−7.7

### 3.7. Relative content of core components

**Figure 7.**
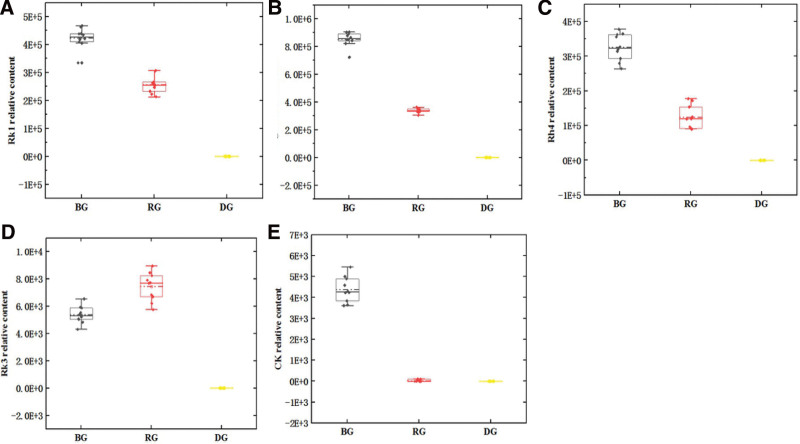
The relative content of the core components in the 3 processed products. (A) ginsenoside-Rk1, (B) ginsenoside-Rg5, (C) ginsenoside-Rh4, (D) ginsenoside-Rk3, (E) compound K.

The relative contents of ginsenoside Rh4, ginsenoside Rk3, ginsenoside Rk1, ginsenoside Rg5, and compound K in different extracted products of 3 kinds of ginseng were compared by box plot (Fig. 7). It was found that these 5 components were mainly concentrated in black ginseng. Almost no raw ginseng was detected, and the content of Rg5 was highest in black ginseng.

## 4. Discussion

With the aging population and the increasing incidence of various risk factors (hypertension, diabetes, obesity, etc.), HF has brought a huge economic burden to the whole of society. Heart failure is the terminal point of most cardiovascular diseases and the leading cause of death in cardiovascular patients. Its high incidence and mortality have become a major public health problem threatening human health.^[5]^ According to previous literature reviews, various ginseng products may have the effect of myocardial protection in the intervention or treatment of HF. Compared with the traditional raw sun-dried ginseng, the content of some rare ginsenosides in black ginseng was increased, but due to the different drying times, it is not certain whether the ginsenosides have changed; at the same time, the information source of active ingredients in TCM database is single and even outdated, which cannot reflect the ingredients in different processed products of ginseng in a comprehensive and timely manner.^[20]^ Therefore, in this paper, UPLC-QE-Orbitrap-MS/MS was used to identify ginsenosides in different processed products of ginseng, and then network pharmacology combined with molecular docking was used to explore the pharmacodynamic substances and mechanism of action in the treatment of HF, supplemented by the relative content of core components in 3 kinds of ginseng products as indicators.

Forty ginsenosides were identified by UPLC-QE-Orbitrap-MS/MS analysis, including ginsenosides Rb1, Rc, Rd, Re, and Rh1.Based on database screening, 124 candidate targets for the treatment of HF were obtained from different derived ginsenosides of ginseng, including 22 core targets such as EGFR, AKT1, ERBB2, STAT3, TNF, ESR1, MTOR, HRAS, MMP9, and PIK3CA. Composition-target-disease-pathway network analysis showed that compound K, ginsenoside Rh4, ginsenoside Rk3, ginsenoside Rk1, ginsenoside Rg5, ginsenoside F4, and other components were associated with more targets. It may be an important component of various derived products of ginseng in the treatment of HF. Rg5 inhibited Drp1 activation and promoted HK-II mitochondrial binding through Akt activation. Rg5 prevented the opening of the mitochondrial permeability transition pore and increased ATP production, thereby enhancing cardiomyocyte resistance to hypoxia/reoxygenation injury.^[21]^ Further mechanistic investigation revealed that Rk3 prevented H/R-induced injury and apoptosis in H9C2 cardiomyocytes via AKT/Nrf-2/HO-1 and MAPK pathways. These observations indicate that Rk3 has the potential to exert cardioprotective effects against H/R injury, which may be of great importance for clinical efficacy in acute myocardial infarction (AMI) treatment.^[22]^ Yang^[23]^ showed that ginsenoside CK intervention could significantly reduce the levels of myocardial necrosis and infarct fiber scar, and reduce the inflammatory factors TNF-α and interleukin in the serum of AMI mice. The secretion of IL-6 increases the secretion of the anti-inflammatory factor IL-10, and this mechanism reduces the inflammatory response by increasing the proportion of regulatory T cells, thereby reducing the injury caused by cardiac remodeling after AMI. The enrichment results of GO and KEGG suggested that the mechanisms of different ginseng derived products in the treatment of HF were mainly related to pathways in cancer, PI3K-Akt pathway, Ras pathway, apoptosis, and mTOR pathway and other pathways, which shows that different derived products of ginseng have the characteristics of multi-target and multi-pathway in the treatment of HF. The results of molecular docking showed that the active components had good binding activity with potential targets, and the results of network pharmacology analysis were reliable to a certain extent.

In conclusion, compared with raw sun-dried ginseng and RG, black ginseng contains more rare ginsenosides, such as ginsenosides Rg3, (20R)-Rg3, Rs3, (20R)-Rs3, Rk1, Rg5, Rh2, CK, etc, while the content of common ginsenosides is very low, such as ginsenosides Rg1, Re, Rb1, Rc, Rb2, Rb3, Rd, etc. However, the content of common ginsenosides is very low, such as ginsenosides Rg1, Re, Rb1, Rc, Rb2, Rb3, Rd, etc. From the research results reported in the literature, it was found that the ginsenosides in black ginseng were mainly rare ginsenosides (Rg5, Rk1, Rg3, Rh4, Rk3, Rg4, Rh1, F4, etc.). However, the contents of common ginsenosides (Rb2, Rb3, Rc, Rd, Rf, Rg2, Rh1, etc.) were relatively low. These results indicated that common ginsenosides were converted into rare ginsenosides during processing.^[24]^ Jin also used the HPLC method to determine the contents of 13 kinds of monomer ginsenosides in white ginseng, RG, and black ginseng. The results showed that the contents of ginsenosides Rg1, Re, Rg2, Rb1, Rc, Rb2, and Rd decreased significantly, but the contents of ginsenosides (20R)-Rg2, Rg3, (20R)-Rg3, Rh1, Rk1, and Rg5 increased significantly during the steaming process of RG and black ginseng.

Through network pharmacology and molecular docking, ginsenosides were found to have the strongest binding force with mTOR in the treatment of HF. mTOR (mechanistic target of rapamycin) is a ~290 kDa, highly conserved atypical filament/threonine kinase and a member of the PIKK family.^[25]^ mTOR is an evolutionarily conserved protein that is widely involved in cell growth and development, regulation of protein synthesis, ribosome biogenesis, T cell fate selection, sensing and integration of upstream signaling pathways of various extracellular stimuli such as growth factors, nutrients, amino acids, starvation, and hypoxia stimuli.^[26]^ mTOR interacts with specific adaptor proteins to form 2 distinct macromolecular functional complexes called mTORC1 (mTOR complex 1) and mTORC2 (mTOR complex 2). mTOR is sensitive to rapamycin and negatively regulates autophagy^[27]^; mTORC2 has no direct effect on autophagy. Partial and selective inhibition of mTORC1 provides cardiac protection in a variety of cardiac pathological conditions, suggesting that pharmacological inhibition of mTORC1 may represent a potential therapeutic intervention target for the treatment of cardiovascular disease. Studies have shown that atorvastatin can improve left ventricular function and remodeling in spontaneously hypertensive rat models, which may be achieved by regulating cardiomyocyte autophagy through the Akt/mTOR signaling pathway. Rapamycin can reduce myocardial hypertrophy, activate cardiomyocyte autophagy and reduce myocardial infarction size by inhibiting mTORC1.Rapamycin plays an anti-cardiac hypertrophy role by activating Akt, promoting protein ubiquitination, and inhibiting cardiomyocyte apoptosis and cardiac fibrosis.^[28,29]^

## 5. Conclusions

This study firstly elucidated the mechanism of multi-component, multi-target and multi-pathway action of various ginseng derivatives in the treatment of HF. It was found that the representative saponins contained in black ginseng have a good prospect in the treatment of HF and cardioprotection based on the mTOR pathway and autophagy, but further pharmacological experiments are still needed to verify this. At the same time, another limitation of this study is that it did not include polysaccharides, proteins, and other macromolecular compounds, which also need further investigation.

## Author contributions

**Conceptualization:** Tingting Dai.

**Data curation:** Tingting Dai.

**Formal analysis:** Tingting Dai.

**Funding acquisition:** Jiyu Gong.

**Investigation:** Tingting Dai.

**Methodology:** Tingting Dai.

**Project administration:** Jiyu Gong.

**Supervision:** Shuying Liu.

**Validation:** Shuying Liu.

**Writing – original draft:** Tingting Dai.

**Writing – review & editing:** Tingting Dai.
